# Ontological representation, modeling, and analysis of parasite vaccines

**DOI:** 10.1186/s13326-024-00307-0

**Published:** 2024-04-25

**Authors:** Anthony Huffman, Xumeng Zhang, Meghana Lanka, Jie Zheng, Anna Maria Masci, Yongqun He

**Affiliations:** 1 Department of Computational Medicine and Bioinformatics, University of Michigan Medicine School, 48109 Ann Arbor, MI USA; 2 College of Literature, Science, and the Arts, University of Michigan, 48109 Ann Arbor, MI USA; 3 Unit for Laboratory Animal Medicine, University of Michigan Medicine School, 48109 Ann Arbor, MI USA; 4 Department of Data Impact and Governance, MD Anderson Cancer Center University of Texas, TX 77030 Houston, USA; 5 Department of Microbiology and Immunology, University of Michigan Medicine School, 48109 Ann Arbor, MI USA

**Keywords:** Parasite vaccine, VIOLIN vaccine knowledgebase, Vaccine Ontology

## Abstract

**Background:**

Pathogenic parasites are responsible for multiple diseases, such as malaria and Chagas disease, in humans and livestock. Traditionally, pathogenic parasites have been largely an evasive topic for vaccine design, with most successful vaccines only emerging recently. To aid vaccine design, the VIOLIN vaccine knowledgebase has collected vaccines from all sources to serve as a comprehensive vaccine knowledgebase. VIOLIN utilizes the Vaccine Ontology (VO) to standardize the modeling of vaccine data. VO did not model complex life cycles as seen in parasites. With the inclusion of successful parasite vaccines, an update in parasite vaccine modeling was needed.

**Results:**

VIOLIN was expanded to include 258 parasite vaccines against 23 protozoan species, and 607 new parasite vaccine-related terms were added to VO since 2022. The updated VO design for parasite vaccines accounts for parasite life stages and for transmission-blocking vaccines. A total of 356 terms from the Ontology of Parasite Lifecycle (OPL) were imported to VO to help represent the effect of different parasite life stages. A new VO class term, ‘transmission-blocking vaccine,’ was added to represent vaccines able to block infectious transmission, and one new VO object property, ‘blocks transmission of pathogen via vaccine,’ was added to link vaccine and pathogen in which the vaccine blocks the transmission of the pathogen. Additionally, our Gene Set Enrichment Analysis (GSEA) of 140 parasite antigens used in the parasitic vaccines identified enriched features. For example, significant patterns, such as signal, plasma membrane, and entry into host, were found in the antigens of the vaccines against two parasite species: *Plasmodium falciparum* and *Toxoplasma gondii*. The analysis found 18 out of the 140 parasite antigens involved with the malaria disease process. Moreover, a majority (15 out of 54) of *P. falciparum* parasite antigens are localized in the cell membrane. *T. gondii* antigens, in contrast, have a majority (19/24) of their proteins related to signaling pathways. The antigen-enriched patterns align with the life cycle stage patterns identified in our ontological parasite vaccine modeling.

**Conclusions:**

The updated VO modeling and GSEA analysis capture the influence of the complex parasite life cycles and their associated antigens on vaccine development.

**Supplementary Information:**

The online version contains supplementary material available at 10.1186/s13326-024-00307-0.

## Background

Parasites are organisms that live on or inside another organism, known as the host, and derive their nourishment from the host. Pathogenic parasites are responsible for causing diseases or disorders in the host, including malaria [1], Chagas disease [2], and livestock disease [3]. There are three main classes of parasites capable of causing disease: protozoa, helminths, and ectoparasites. Among these, protozoans are unicellular microorganisms with diverse life stages. Protozoan parasites can be classified into four clades: Mastigophora (flagellates), Sarcodina (amoeba), Apicomplexa (non-motile protozoans in their adult stage), and Ciliophora (ciliates) [4]. Traditional treatment for parasite-caused diseases has relied on specific drug treatments targeting symptoms of the disease instead of generating immune memory through vaccines [5]. However, recent years have witnessed successful clinical trials of parasite vaccines [5, 6]. This success can be attributed to traditional vaccine design focusing on pathogens with a single life stage for immunization.

**Fig. 1:**
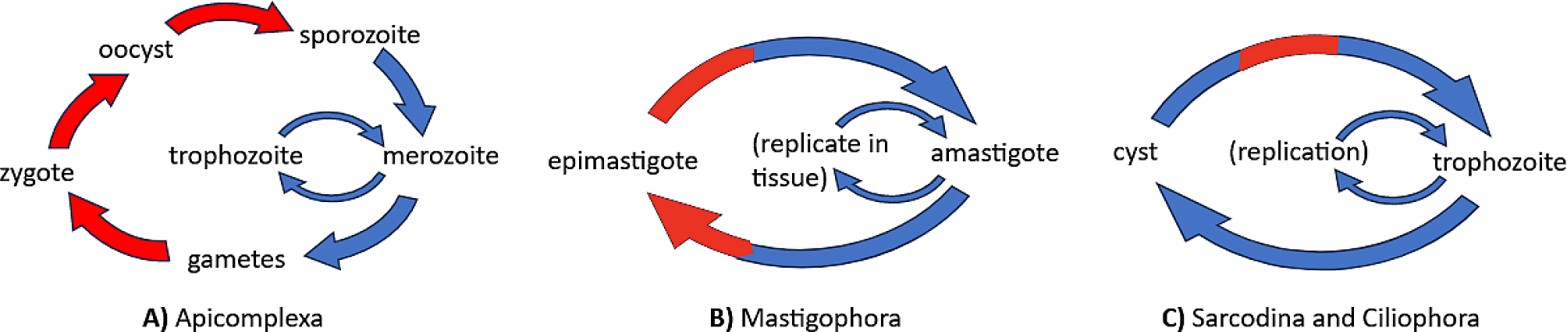
The complicated life cycle of protozoan parasites. The red and blue colors represent the life stages that occur in the vector and host of the parasite, respectively. (**A**) Apicomplexan parasite life stages are clearly identified in their reproductive cycle. The exception is the trophozoite stage, which refers to the stage where it draws nutrition or energy from an explicit host. (**B**) Mastigophora parasite life stages are based on the state of their flagellum. (**C**) Ciliophora and Sarcodina parasites have similar life stages that alternate from a cyst (usually made of eggs) to trophozoite. Simplified life cycles were referenced from the CDC [4]

Parasitic protozoans have evolved multiple distinct phenotypes as part of their developmental cycle (Fig. 1). Protozoan parasites alternate between distinct life stages adjusted to their preferred hosts [7]. Apicomplexan parasites (Fig. 1A), such as the malaria parasite, typically exist within a host during the diploid life stage or as a haploid in a vector. For example, the sporozoite stage of *Plasmodi* initially targets human hepatic cells. The *Plasmodium* then develops merozoites that target erythrocytic cells. The merozoites either continue to develop into trophozoites, which cause the malaria disease process, or mature into gametocytes for the vector to ingest. While within mosquitoes, the gametocytes will eventually undergo mitosis to produce sporozoites to repeat their life cycle [7]. A simpler life cycle can occur in other parasites. Mastigophora parasites (Fig. 1B) alternate between a mobile epimastigote stage when outside of a host. However, once within a host, the epimastigote can burrow and replicate within the tissue of the host. The last two main categories of parasites, Sarcodina and Cilophora, have a simpler, generic life cycle. These parasites alternate as cysts that can survive outside the host before producing trophozoites, which burrow into the target tissue [8].

Vaccines for infectious diseases are processed materials designed to elicit an immune response against a pathogen(s) using antigens. Antigens used in vaccines are typically derived from the target pathogens or the disease-causing agents. These antigens can include various forms, such as live, attenuated, inactivated whole pathogenic organisms, or subunit components of the pathogens, depending on the type of vaccine and the specific strategy used to induce an immune response [9]. When a vaccine is administered, it stimulates the immune system to recognize the specific antigen(s) present, triggering the activation of immune cells and pathways. This activation leads to the production of antibodies and the formation of memory cells [10]. To enhance the immune response to antigens and improve their effectiveness, adjuvants are often added to vaccines [11]. To support vaccine data standardization and integration, the Vaccine Ontology (VO) currently serves as a specialized ontology aiming to standardize the representation and organization of vaccine and vaccine-related knowledge [12].

Parasite vaccines have been traditionally hard to design for. Using malaria as an example, the development of Mosquirix (P. falciparum RTS, S vaccine), the first licensed malaria vaccine, took 34 years of development and testing before being approved for use in 2021 [13]. Except for specific vaccines used for livestock, parasite vaccines for humans remain elusive. In contrast, parasite chemical drugs aimed to alleviate or counter a parasite in a host represent a more mature technology. The first synthetic anti-malaria drug, Quinine, was successfully synthesized in 1944 [14] and targeted the erythrocytic life stage. Since then, multiple malaria drugs have been developed as either derivatives (chloroquine) or targeting different life stages (sporozoites or ookinetes) [15]. In both these cases, parasite vaccines and chemical drugs succeeded by targeting specific life stages instead of attempting to fully cover the entire parasite life cycle. With new technologies being developed, more efforts have been devoted in recent decades to studying and developing various types of parasite vaccines.

Ontology, as a system, organizes and categorizes knowledge within a specific domain, creating a uniform vocabulary of terms and relations representing entities and relations among the entities in the domain. This standardized representation enables the utilization of consistent terminology across different data sources and facilitates the extraction of hidden knowledge from large-scale data through meaningful annotations that computers can understand. Ontologies are organized by specific domains. Within the context of this paper, we primarily utilize the Vaccine Ontology (VO) [12] and Ontology for Parasite Lifecycle (OPL) [16]. The VO models all components of vaccines, such as vaccine components, vaccine targets, and host responses to vaccines. By default, the VO framework assumes that a vaccine that works against a pathogen (e.g., SARS-CoV-2) would work against all strains of the pathogen. However, parasites have complex and distinct life stages that conflict with the current VO assumption. Therefore, a new VO modeling framework for parasite vaccines is needed. The OPL, on the other hand, fully models the different life stages that occur in parasites, including the targeted host of a specific parasite. As such, reutilizing OPL terms to represent parasite vaccines in VO is a natural fit.

We collected parasite vaccines that immunize against twenty-three parasite species that are in different stages of research, clinical trials, or approval. Such information is collected and stored in VIOLIN; however, many unique characteristics of parasites were not appropriately represented within our VO. To address these issues, we have updated VO with a new design pattern to handle the complexities of parasite vaccines. Using the final list of collected parasite antigens, we have also analyzed gene set analysis to identify more common patterns for parasite antigens. Finally, we show how to query the information within VIOLIN. As such, our work focuses on systematically representing and analyzing parasite vaccines, primarily for mastigophorans and apicomplexans. By employing ontology to represent the differences in parasite life stages, we aim to further analyze patterns in protective antigens from experimentally verified studies. This approach will contribute to the advancement of successful parasite vaccines.

## Methods

### Parasite vaccine collection and annotation in VIOLIN

A literature search was conducted using PubMed and clinicaltrials.org to identify relevant information on parasite vaccines. Manual annotation of the collected data was performed, and the information was integrated into the VIOLIN knowledgebase (https://violinet.org). The collection process included capturing details such as vaccine type, protective antigens, adjuvants, vectors, experimental or clinical trial data (subjects, protocols, immune responses, efficacy), and more. The annotation process was guided through the use of the VO.

### Ontology modeling and term collection

A literature search was conducted to identify common patterns in parasite life stages, hosts, and vectors. Protective antigens were mapped to specific parasite life stages using terms in NCBITaxonomy (NCBITaxon), OPL and VO. This information was used to expand the VO by importing terms from OPL using Ontofox [17]. New classes were added in VO through Ontorat [18] following an ontology design pattern. New object properties were generated through the use of Protégé [19]. This step aimed to create an updated VO ontology that accurately represents the parasite vaccines with different life stages of parasites.

### Gene ID mapping

NCBI gene IDs for the annotated protective antigens were extracted from the NCBI database (https://www.ncbi.nlm.nih.gov/gene). The NCBI gene IDs were initially searched from the published literature or clinical trial websites. If the gene ID of the antigen was not available, a search was conducted in the NCBI database using the antigen name. In cases where no results were found using the antigen name, the antigen sequence was searched against the NCBI database using the Basic Local Alignment Search Tool (BLAST). The obtained NCBI gene IDs were then added to the VIOLIN database (https://violinet.org/index.php) based on the mapping result.

### Web-based VIOLIN query and online analysis of parasite vaccines

The VIOLIN website query was used to gather all summary statistics about parasite vaccines and antigens. This was also done to gather information on parasite vaccines and antigens that were previously added to VO. Once the collection of the parasite vaccines and the corresponding NCBI gene IDs of protective antigens were obtained, the list of gene IDs was submitted to the Database for Annotation, Visualization and Integrated Discovery (DAVID) [20]. Gene set enrichment analysis (GSEA) was performed using DAVID’s functional annotation tool, and the annotation results for each parasite genome were collected (Supplemental Table 2). A False Discovery Rate (FDR) of 0.05 or less was used to identify statistically significant pathways. This analysis was conducted individually for each species of parasite vaccine.

## Results

### VIOLIN parasite vaccine collection and annotation

In total, VIOLIN includes 258 parasite vaccines directed against 23 parasite pathogens (Supplemental Table 1). We have added 181 new parasite vaccines to VIOLIN since 2022. From all the parasite vaccines collected, 182 parasite antigens were mapped to 140 NCBI gene IDs for further analysis. Of these 258 parasite vaccines, 86 are Mastigophoran vaccines, with a total of 51 Mastigophoran protective antigens across eight species. From the 172 Apicomplexan vaccines for 15 parasites (Supplemental Table 1), 131 Apicomplexan protective antigens were identified. There are 12 licensed vaccines for non-humans: 8 for *Eimeria spp.*, 2 for *Neospora caninum*, 1 for *Trypanosoma cruzi*, and 1 for *Leishmania donovani*. Otherwise, most parasite vaccines remain in the clinical trial or research stage.

Table 1 provides a list of parasites with at least ten vaccines curated in VIOLIN. Over half of the vaccines collected were for three species: *Toxoplasma gondii* (58), *Plasmodium falciparum* (42), and *Trypansoma cruzi* (34). At a genus level, *Plasmodium* vaccines account for a quarter of vaccines, with 67 vaccines.

**Table 1:** A list of parasites with at least ten vaccines curated in VIOLIN. VIOLIN sorts vaccines by disease, and as such combines *Plasmodium* and *Eimeria* species into single entry

Pathogen Name	Disease	# of vaccines	# of Licensed Vaccines	# of mapped vaccine antigens
*Plasmodium spp.*	Malaria	67	0	54
*Typanosoma cruzi*	Chagas disease	34	1	27
*Leishmania donovani*	Visceral leishmaniasis	17	1	16
*Toxoplasma gondii*	Toxoplasmosis	58	0	26
*Leishmania major*	Cutaneous leishmaniasis	13	0	15
*Eimeria spp.*	Coccidiosis	11	8	1
*Neospora caninum*	Neosporosis	11	2	10
*Schistosoma japonicum*	Schistosomiasis	10	0	8

**Fig. 2:**
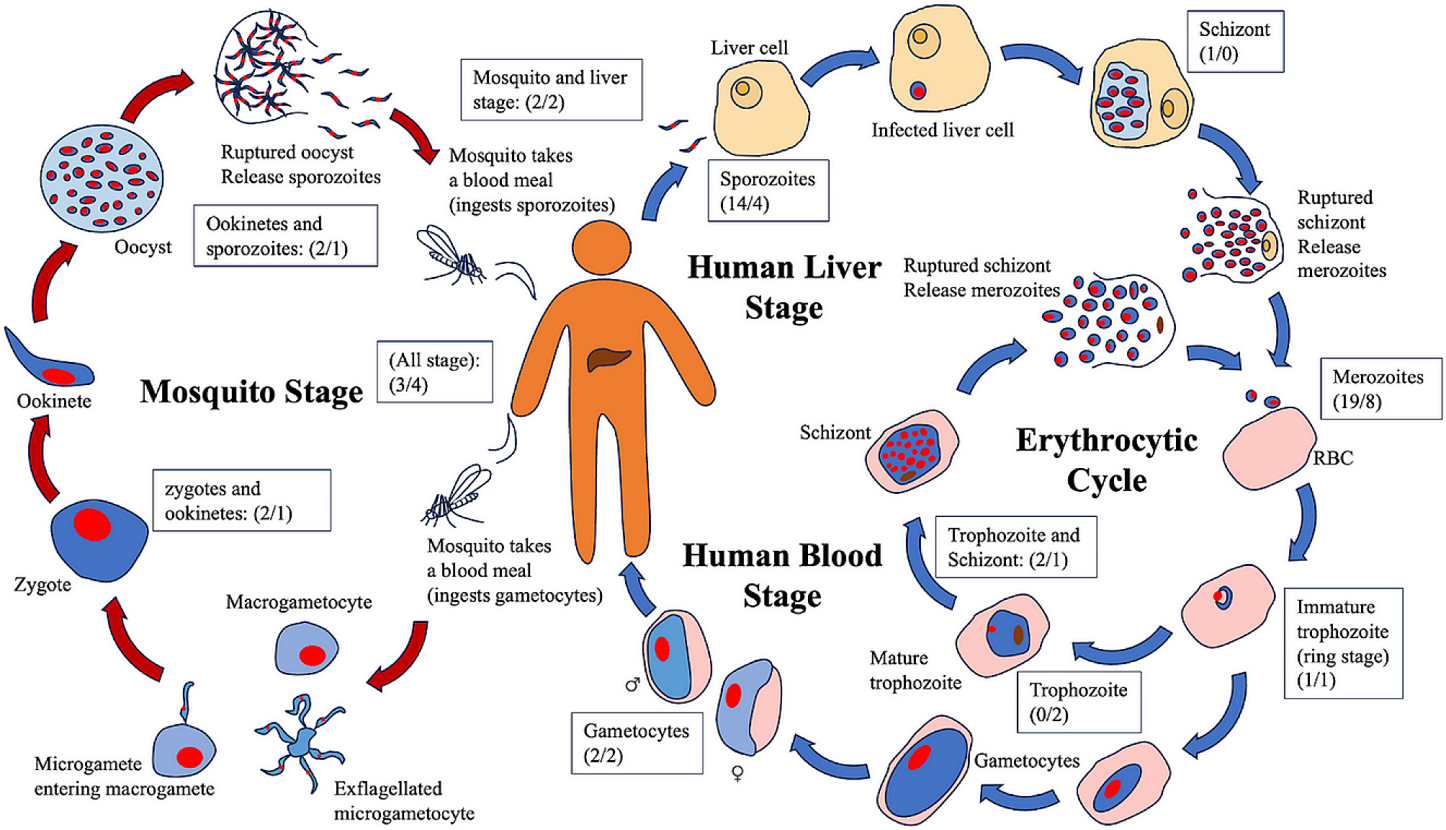
*Plasmodium falciparum* life cycle and vaccines at different stages. The numbers of annotations of vaccines / antigens in VIOLIN relabeled beside corresponding life stages. *P. falciparum* parasites (blue cells with red nuclei) enter the human body when a parasite-carrying mosquito takes a blood meal. The sporozoites infect human liver cells (yellow), in which the parasite replicates and eventually releases merozoites. The merozoites infect human red blood cells (red), and the ring-stage parasites either enter the erythrocytic cycle for asexual reproduction or develop into gametocytes. The gametocytes are taken by mosquitos during another blood meal and sexually reproduce [21]. The red arrows represent the mosquito stage, whereas the blue arrows represent the human stage. Figure and life cycle adapted from NIH.gov

Fig. 2 shows *P. falciparum* life cycles and vaccines targeted to specific life cycle stages. Due to the difficulty in generating effective *P. falciparum* vaccines, many vaccines target different lifecycle stages. Most vaccines target *Plasmodium* merozoites, which is responsible for inducing malaria disease. The second most frequently targeted stage is the *Plasmodium* sporozoite, which is the initial life stage that can infect a human or mammalian host. Despite this, most parasites have vaccines that target different parasite life stages. This prompted a need for the VO to account for the different parasite life stages as part of its high-level design.

### Ontology modeling of parasite vaccines

**Fig. 3:**
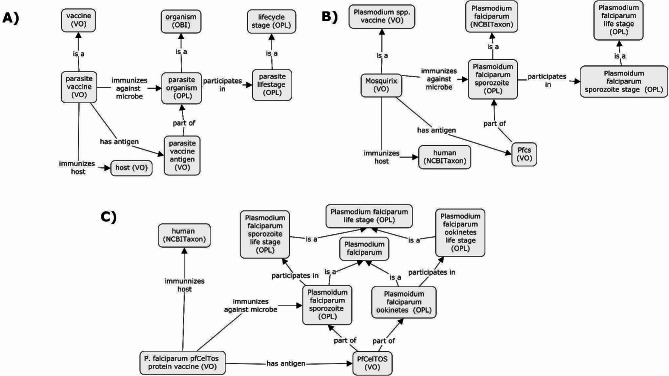
ODP of parasite vaccine within VO. (**A**) The Ontology design pattern (ODP) of transmission blocked parasite vaccines within VO. Currently all blocked transmission vaccines in VO are also parasite vaccines. (**B**) Representation of parasite antigen PfCelTOS (*P. falciparum* cell traversal in sporozoite and ookinetes) and (**C**) Pfcs (*P. falciparum* circumsporozoite protein) within VO. Pfcs is currently used in the only licensed malaria vaccine despite only targeting one life stage. PfCelTOS is found in multiple life stages and thus may have a greater range of targets

We have designed a new VO Ontology Design Pattern (ODP) for the new parasite vaccines shown in Fig. 3A). The primary change for this design pattern is the inclusion of parasite life stages. VO traditionally utilizes ‘immunizes against pathogen’ some ‘organism’ for vaccine design. However, due to the complexity and specificity of parasites, parasite vaccines instead ‘immunizes against pathogen’ against some parasite organisms at a specific life stage. Following OPL, each parasite organism at a specific life stage is a subclass of the parasite. Using this ODP, we updated the VO with 179 new vaccines and 15 new vaccine categories related to parasite life stages. We have also added 57 new terms relating to vaccine antigens. We have imported 356 new terms from OPL to represent different life stages. Additionally, the representation of multiple parasite stages can be used to clarify the intended target of a specific parasite vaccine (Fig. 3B and C) by linking the vaccine antigen to the parasite at a specific life stage. This is done as specific antigens are expressed in specific life stages.

Using the above ODP, the malaria vaccine Mosquirix (P. falciparum RTS, S/AS01) is represented in VO with the following axioms:

‘is a’ ‘Plasmodium falciparum vaccine’

‘has part’ some ‘protein of pathogen organism as vaccine component’

‘has vaccine antigen’ *some* ‘P. falciparum CSP’

‘has vaccine adjuvant’ *some* ‘liposome-based vaccine adjuvant’

‘immunizes against pathogen’ *some* ‘Plasmodium falciparum sporozoite’

‘immunizes host’ *some* ‘Homo sapiens’

**Fig. 4:**
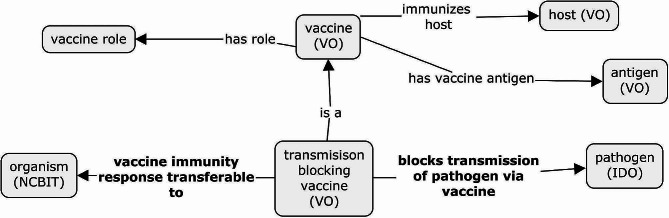
DL query of*Plasmodium falciparum*merozoite vaccine in VO. The use of the OR for vaccine antigen or has part is to fully cover vaccines that use the antigen or use the whole organism as part of the vaccine. Additional OR statements can be used to search for vaccines that occur in multiple life stages

The primary use of including life stages is to identify what stage a vaccine would be most effective and to infer additional life stages that the vaccine can target. For example, as the merozoite stage is responsible for the malaria disease process within humans, we can construct a query to generate a list of possible vaccines that can be used to target a specific parasite life cycle stage. For instance, the PfCelTOS vaccine can potentially target the sporozoite and merozoite stages due to the PfCelTOS antigen occurring in those two life stages (Fig. 3C). Figure 4 shows the construction for this query. This can be expanded further to look for vaccines that can target multiple parasite life stages. The vast majority of the *P. falciparum* vaccines do not show overlap between multiple life stages.

### Ontological modeling of transmission-blocking vaccines against parasite life stages

Parasite vaccine development has explored alternative methods to control the population of parasite pathogens beyond traditional vaccine design. One such approach is the use of transmission-blocking vaccines, such as PFS25/28 in Matrix-M [22]. These vaccines aim to interfere with the transmission of the pathogen from the vaccine target to other hosts. Currently, transmission-blocking vaccines are employed to prevent the spread of *Plasmodium* spp. from mosquitoes, which occurs when mosquitoes feed on infected humans. These transmission-blocking vaccines work by using the immune response generated by a human to interfere with the development of a malaria parasite zygote within the Anopheles mosquito gut [23].

**Fig. 5:**
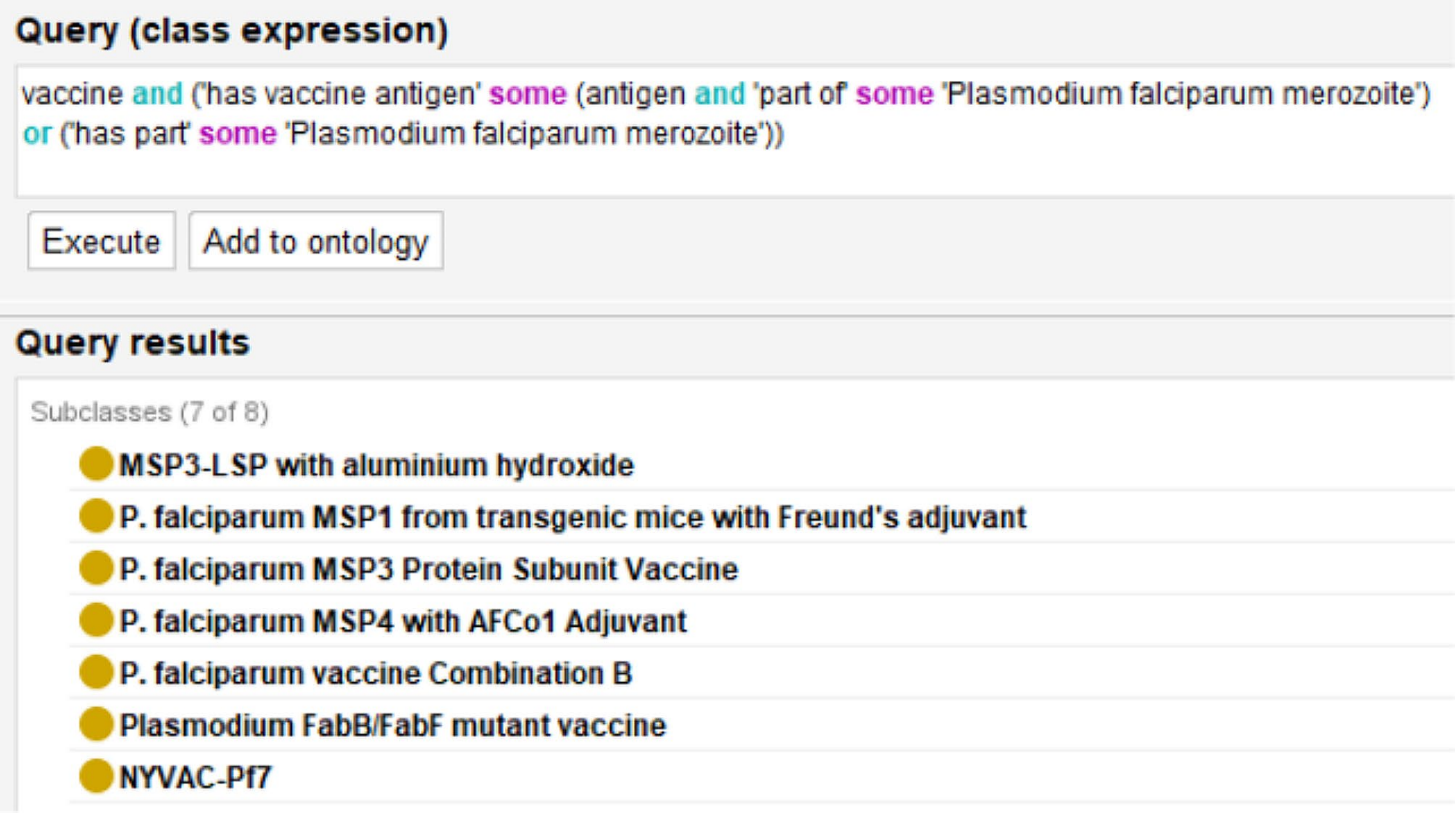
ODP for Transmission Blocking Vaccines. The primary expansion for transmission-blocking vaccines is the inclusion of two new relationships (bolded). The use of ‘blocks transmission of pathogen via vaccine’ is used as a more specific variation of ‘immunizes against pathogen’

We define a ‘transmission-blocking vaccine’ as a vaccine that prevents or reduces the transmission of a disorder within a target host or among a population by inducing or modifying specific adaptive immune responses against the antigens present in the vaccine. The modeling of these relationships is shown in the VO as part of Fig. 5. Within the concept of transmission-blocking vaccines, we introduce two properties.

The first is ‘blocks transmission of pathogen via vaccine’, which represents the relationship between a vaccine (material entity) and an organism with a pathogen role (material entity). Def: “A relationship between a vaccine and a pathogen where the vaccine immunizes against some parasite by blocking the transmission of the parasite to additional hosts or vectors.”

This relationship implies that the material entity participates in a process that results in the population of the pathogen (e.g., parasite) being blocked from transmitting to additional hosts or vectors. A vaccine that ‘blocks transmission of pathogen via vaccine’ is referred to as a ‘blocked transmission vaccine’. The ‘blocks transmission of pathogen via vaccine’ is asserted as a child object property of ‘immunizes against pathogen’.

The second object property is ‘vaccine immunity response transferrable to,’ which describes the transfer of the vaccine-induced immune response to a non-vaccine host. We define ‘vaccine immunity transferrable to’ as follows:

Def: ‘A relationship between a vaccine X and organism Y where the vaccine X generates an immune response in a vaccinated organism and the immune product (e.g., antibody) can be transferred to another organism Y.’

We note that this relationship should be paired with the normal ‘immunizes host’. Additionally, it serves as a shortcut property to represent the following set of statements:

If vaccine X immunizes host A, and A produces antibody Z, and Z is transferred to organism Y, then X ‘vaccine immunity response transferable to ‘organism Y.’

For example, ‘PFS25/28 in Matrix M’ has been identified as an effective transmission-blocking vaccine for malaria [23]. The following VO axioms logically define the vaccine (the underlined text shows an inferred parent vaccine term for the vaccine).

PFS25/28 in Matrix M

‘is a’ ‘transmission-blocking vaccine’

‘is a’ ‘Plasmodium falciparum vaccine’

‘blocks transmission of pathogen via vaccine’ s*ome* ‘‘Plasmodium falciparum gametocyte’

‘has role’ *some* ‘subunit vaccine role’

‘has vaccine antigen’ *some* ‘Plasmodium falciparum Pfs25’

‘immunizes against pathogen’ *some* ‘Plasmodium falciparum gametocyte’

‘immunizes host’ *some* ‘homo sapiens’

‘has vaccine adjuvant’ *some* ‘Matrix-M vaccine adjuvant’

‘vaccine immunity response transferrable to organism’ *some* ‘Anopheles <genus>’

### Gene ID mapping and enriched profiles of protective antigens

From our antigen list, we found 146 gene IDs for 22 pathogens, which were further analyzed for significantly enriched features. Supplemental Table 2 contains the list of significant features found for the protective antigens of each parasite. Only two terms from the 40 Mastigophoran gene IDs was statistically significant: 2 of 9 analyzed *T. cruzi* antigens annotated term ‘Motile Cilium’ (FDR = 4.8E-02), and 2 of 9 analyzed *T. cruzi* antigens annotated term ‘Chagas disease’ (FDR = 3.5E-02) (Table 2), indicating slight similarities in the functions of these antigens in cellular movement or disease pathology. On the other hand, 106 mapped Apicomplexan gene IDs revealed 40 statistically significant terms (Supplemental Table 2), most of which were from *P. falciparum* or *T. gondii* (Table 2).

Strong correlations were found between signal sequence and protective antigens of many Apicomplexan species. The UniProt term ‘Signal’ was enriched for the following numbers of antigens: 19 of 34 analyzed *T. gondii* antigens (FDR = 9.6E-07), 15 of 22 analyzed *P. falciparum* antigens (FDR = 1.4E-06), and 6 of 7 analyzed *B. bovis* antigens (FDR = 2.3E-03) (Supplemental Table 2).

In addition, our result showed that some antigens were involved in disease pathology, especially antigens of *Plasmodium, Trypanosoma*, and *Toxoplasma*. ‘Malaria’ and ‘Toxoplasmosis’ terms under the KEGG Pathway category frequently appeared as statistically significant annotations in the GSEA result (Table 2). However, the antigen numbers of these terms were lower compared to the signal sequence term, indicating that many protective antigens were not included in the KEGG pathway of these diseases. For example, only 7 of 34 analyzed *T. gondii* antigens annotated term ‘Toxoplasmosis’ (FDR = 4,7E-06), and only 9 of 22 analyzed *P. falciparum* antigens annotated term ‘Malaria’ (FDR = 4.0E-03). Although there were some correlations between the immunogenicity of the protein and the disease pathology, many antigens were not shown to be disease-related.

Besides the similarities, distinctions between the GSEA results of different parasite species were noticeable when looking into the annotations under each parasite genome background, especially for *T. gondii* and *P. falciparum*. 8 of 11 statistically significant *T. gondii* gene functions were ATP or Kinase related (Supplemental Table 2). This result showed a strong correlation between *T. gondii* protective antigens and phosphorylation, indicating that most *T. gondii* antigens are internally located and involved in infection. On the other hand, *P. falciparum* protective antigens annotated several statistically significant terms related to membrane and protein binding (Supplemental Table 2). For example, the FDR for ‘Plasma Membrane’ was 9.9E-13 (Table 2), while the FDR for ‘Anchored Component of Plasma Membrane’ was 3.4E-04 (Table 2). In addition, *P. falciparum* protective antigens also showed a strong correlation with ‘Entry to Host’ (FDR = 2.5E-07). These results indicated that *P. falciparum* protective antigens are likely to be membrane proteins that participate in host invasion.

**Table 2:** Most significant GSEA clusters of Apicomplexans (*P. falciparum* and *T. gondii*) & Mastigophorans (*T. cruzi*). A full list of enrichment terms can be found in Supplemental Table 2

Species Gene Set	GO / UniProt Enrichment Term	# of antigens	Percentage	*p*-value	FDR
*T. gondii*	Signal	19	25.3	1.6E-07	9.6E-07
*T. gondii*	Toxoplasmosis	7	9.3	3.9E-07	4.7E-06
*T. gondii*	Protein kinase	7	9.3	3.9E-05	1.1E-03
*P. falciparum*	plasma membrane	13	17.3	2.5E-14	9.9E-13
*P. falciparum*	Signal	15	20.0	2.3E-07	1.4E-06
*P. falciparum*	entry into host	9	12.0	9.9E-09	2.5E-07
*P. falciparum*	Anchored component of plasma membrane	4	5.3	4.4E-05	3.4E-04
*P. falciparum*	Malaria	9	12.0	4.0E-04	4.0E-03
*T. cruzi*	motile cilium	2	5.3	9.6E-03	4.8E-02
*T. cruzi*	Chagas disease	2	5.3	3.5E-02	3.5E-02

### VIOLIN query of parasite vaccines

**Fig. 6:**
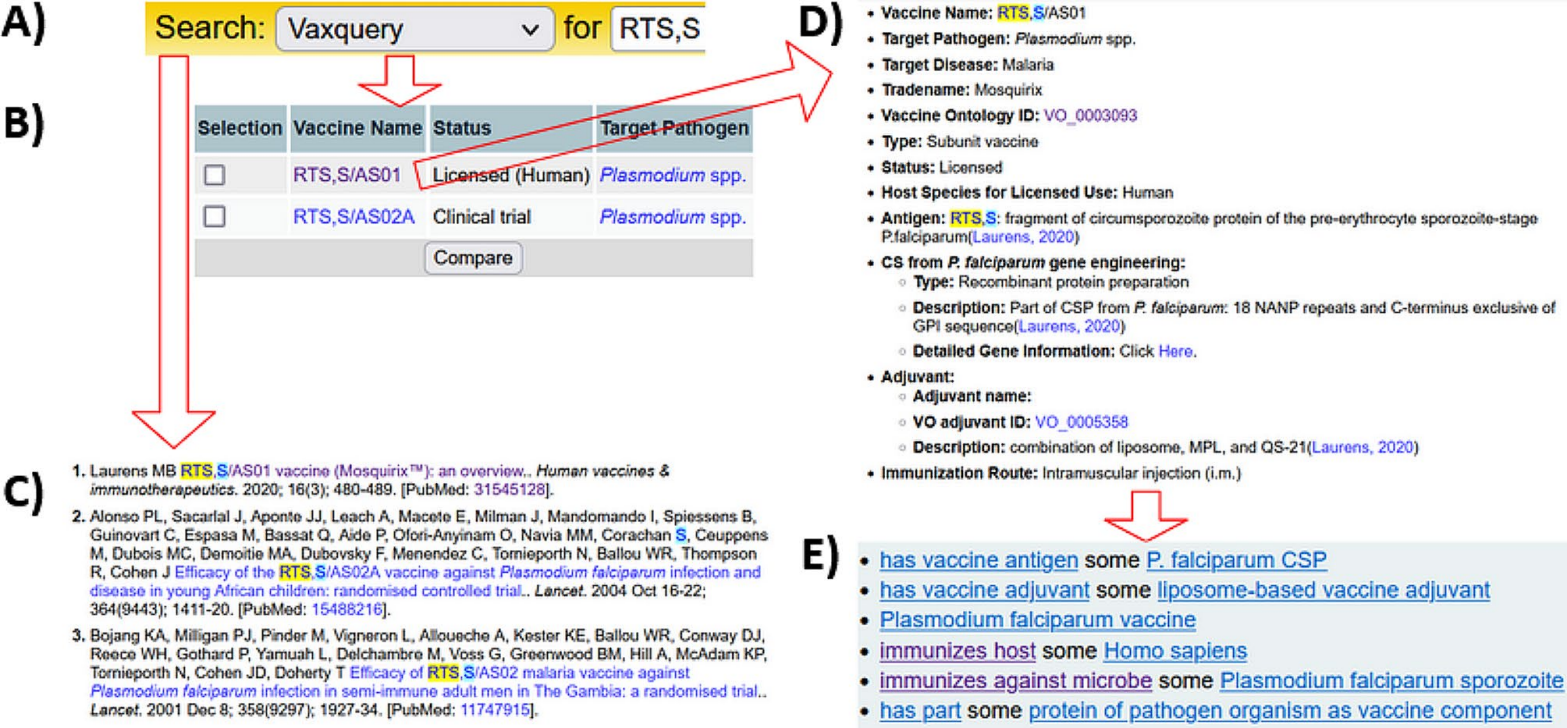
VIOLIN web query for the licensed vaccine. (**A**) Vaxquery can be used to directly query for all entries that contain the query substring. The results for “RTS, S” query are used to find a collection of (**B**) all vaccines and (**C**) all sources that match the criteria. The RTS, S vaccine has two formulations, with RTS, S/AS01 being used for Mosquirix. The query “RTS, S” returns no results for pathogens or gene names. (**D**) Detailed vaccine information can be found when clicking the vaccine name (shown in red arrows). (**E**) The selection of a Vaccine Ontology ID can be used to click a link to Ontobee (https://ontobee.org) to find all axioms found for a term within VO for the vaccine or vaccine components. The multiple RTS, S vaccines are due to the differences in vaccine adjuvants. Red arrows show the direction of links and what information is produced

The VIOLIN knowledgebase includes a system to query and integrate all information about a specific vaccine. An example of such a query was conducted by searching the primary antigen for the Mosquirix vaccine, RTS, S (Fig. 6). The VIOLIN Vaxquery program can be accessed via a search bar to search for an entry by name (Fig. 6A) or can be expanded to specify additional criteria for vaccine selection. Vaxquery returns all matches within VIOLIN for that name in vaccines (Fig. 6B), pathogens, vaccine genes, or references (Fig. 6C). Each vaccine within VIOLIN (Fig. 6D) is described following the standardization of terms and relationships using VO. In addition, each term in VIOLIN has an entry to include its corresponding VO ID, which can be used to link to additional annotations and axioms for this vaccine. This allows reasoners to aid in research or queries. Additionally, VIOLIN links to Ontobee (Fig. 6E), a website that tracks the utilization of ontology terms across multiple ontologies. Altogether, VIOLIN integrates knowledge from multiple sources to provide comprehensive information on vaccines.

## Discussion

In this study, we focused on annotating, modeling, and analysis of Mastigophoran and Apicomplexan vaccines. Our study contributes in multiple ways. First, we annotated parasite vaccines (esp. Mastigophoran and Apicomplexan vaccines) by gathering information from published literature and clinical trial websites. In total, there are 258 Mastigophoran and Apicomplexan vaccines annotated in VIOLIN, which include 51 Mastigophoran protective antigens and 131 Apicomplexan protective antigens. All the parasite vaccine information was added to VO, including our newly added 179 parasite vaccines, 57 vaccine antigens, 15 new vaccine categories, and 4 ontological object property relationships. We also imported 356 terms related to parasite life stages from OPL. Furthermore, we performed GSEA analysis using DAVID functional annotation to identify patterns in protective antigens. A total of 42 statistically significantly enriched terms were identified, two of which (i.e., motile cilium and Chagas disease) were associated with *T. cruzi* (a Mastigophoran parasite), while the others were associated with Apicomplexan parasites *P. falciparum* and *T. gondii*.

The primary change to VO has been the development of ontological models to cover the representation of unique features of parasite vaccines. The primary modeling issue was that parasites exhibit multiple life stages that are phenotypically distinct and target distinct locations for their parasitism. In comparison, while there has been ongoing research to account for targeting pathogenic variants of commensal bacteria (e.g., *E. coli)*, bacterial vaccines assume a phenotypic uniformity as part of the organism. Viral vaccines are assumed to, in general, be effective against all instances of the virus for which the vaccines are designed. For example, the Coronavirus Infectious Disease Ontology (CIDO), a biomedical ontology focused on coronaviruses, identified specific variants of coronaviruses by listing strains as subclasses for specific viruses [24]. By listing a vaccine (e.g., Moderna COVID-19 vaccine) as a COVID-19 vaccine in VO, the VO assumes that the Moderna vaccine is used for immunizing hosts against the infection of SARS-CoV-2 coronaviruses, and here the VO does not differentiate different SARS-CoV-2 variants. Different from the relatively simple situations in bacterial and viral vaccines, parasites have complex life cycle stages, and parasite vaccines are usually designed to target specific stages. To our knowledge, this work represents the first ontological modeling of vaccines by considering the microbial life cycle complexity.

The modeling of transmission-blocking vaccines includes the representation of a third organism outside of the usual host and pathogen that all vaccines have as participants. While currently, all transmission-blocking vaccines are parasite vaccines, there is current research on the viability of drugs to limit the transmission of malaria [25]. As such, specifying two relations, one for a general material entity and another specifically for vaccines, was warranted.

Based on our GSEA analysis, distinctive properties were identified in the protective antigens of different Apicomplexan parasites, particularly *T. gondii* and *P. falciparum*. A strong correlation was found between signal sequence and Apicomplexan protective antigens (Table 2, Supplemental Table 2). Signal sequences guide ribosomes to the endoplasmic reticulum (ER) and facilitate cotranslational insertion, leading to the protein’s localization from the cytoplasm to the extracellular space [26]. This result suggests that many immunogenic proteins discovered to date are likely to be secreted proteins or membrane components. This observation aligns with the involvement of both secreted proteins and membrane proteins in the host cell invasion [27] and adaptation processes [28] of Apicomplexan parasite. Meanwhile, a large proportion *of P. falciparum* protective antigens are membrane proteins with anchored components (Table 2).

The differences in enriched antigen patterns can be attributed to the variations in the life cycle and host cell invasion strategies of these parasites. Being able to affect all warm-blooded animals as intermediate hosts [29], *T. gondii* has strong invasion and adaptation capabilities to survive in various host environments that are mainly provided by the secreted proteins [30, 31]. *T. gondii* microneme proteins are crucial for the gliding motility of the parasite and the adhesion of the parasite to the host cell [30]. *T. gondii* rhoptry proteins affect host cell physiology by modulating host cell signaling pathways [31] and take part in parasitophorous vacuole formation [30], both of which make the host cell environment more suitable for parasite survival [31]. Most of the *T. gondii* rhoptry proteins are known to be kinases or pseudokinases [31], which corresponds to our result that multiple *T. gondii* protective antigens are phosphorylation-related. On the other hand, *P. falciparum* has a much more restricted host range and a much more complicated life cycle compared with *T. gondii*. Different surface proteins are involved in the attachment of *P. falciparum* to specific host cells at different infectious life stages. For example, *P. falciparum* circumsporozoite proteins (CSP) cover the surface of *P. falciparum* during the developmental life stages of the parasite [32]. The two conserved motives of CSP play important roles in sporozoite invasions by interacting with host cell molecules from mosquito salivary glands and human hepatocytes, respectively [32]. *P. falciparum* merozoite surface proteins (MSPs) are involved in the initial attachment of *P. falciparum* merozoite to the red blood cell (RBC). MSPs form the MSP complex [33], and the MSP1 at the binding sites interacts with RBC surface proteins such as glycophorin A (GPA) and Band 3 [32]. Most MSPs were initially anchored to or loosely bound to the parasite surface [33], which corresponds to our result that a lot of *P. falciparum* protective antigens are membrane proteins or have anchored components. Therefore, analysis of these antigen patterns will help our understanding of parasite vaccine mechanisms and support further rational vaccine design.

There are many directions in future parasite vaccine research. One direction is to expand the search for more antigens and broaden the analysis to encompass parasitic vaccines as a whole rather than focusing on a specific subphylum. One current limitation of our GSEA analysis is the small set of annotated gene IDs available for many pathogens. As a result, we were unable to map gene IDs for all protective antigens collected, especially for mastigophoran antigens. Additionally, the limited number of protective antigens found for several parasite species hindered our ability to identify statistically significant terms. As more parasite vaccines are now advancing through clinical trials, such as the R21/matrix-M malaria vaccine [34], the number of known parasite protective antigens will increase. Furthermore, we are developing new machine learning or reverse vaccinology methods [35, 36] to support parasite vaccine design. The newly collected and annotated parasite vaccine results will provide good training data for our method development.

## Conclusion

This paper reports our expanded collection of 258 parasite vaccines against 23 protozoan species, the inclusion of these vaccines to the Vaccine Ontology (VO), ontological modeling of parasite vaccines, and a gene set enrichment analysis for finding features significantly associated with parasitic vaccine antigens. A novel contribution is the updated VO design to model and represent parasite life stages and transmission-blocking vaccines. By integrating the new parasite vaccines into the VO, we were able to capture the influence of different parasite life cycles on vaccine development. We also identified gaps in the representation of parasite diseases and demonstrated the potential associations between sequence features and effective parasite vaccine antigens through our gene set enrichment analysis (GSEA). While this GSEA analysis is largely limited to apicomplexan vaccines, it still provides a source for future investigations. Such GSEA analysis provides results complementary to our VO ontology modeling. As such, we demonstrate that with our updates to Vaccine Ontology, we can establish new, meaningful connections for parasite and vaccine research.

### Electronic supplementary material

Below is the link to the electronic supplementary material.

Supplementary Material 1

Supplementary Material 2

## Data Availability

The VIOLIN knowledgebase used to store the vaccine data is available at https://violinet.org/. This knowledge base contains information on the vaccines and antigens used for this analysis. Vaccine Ontology is available at https://github.com/vaccineontology.

## Abbreviations

OPL: Ontology of Parasite Lifecycle
VO: Vaccine Ontology
SARS-CoV-2: Severe acute respiratory syndrome coronavirus
VIOLIN: Vaccine Investigation and Online Network
NCBITaxon: NCBI Taxonomy
DAVID: Database for Annotation, Visualization, and Integrated Discovery
FDR: False Discovery Rate
GSEA: Gene Set Enrichment Analysis
